# Saccharine

**Published:** 1888-10

**Authors:** 


### SACCHARINE.

This newly discovered quintessence of sweetness is now being largely used in the arts and medicine, and gives evidence of, in time, becoming a formidable competitor in the open market with sugar. It has already, in some places, supplanted glucose in the manufacture of confectionary. It is extensively used as a sweetening agent in the manufacture of beer by the Germans, so much so that the English papers are advocating its use in England, as the German article, so made, can be sold much cheaper in England than the home-made article, and is thereby undermining them in their own market. Its place in our pharmacopœia is not as yet established. It does not appear to have any effect upon the animal economy, and it was this supposed characteristic that caused it to be hailed as an excellent agent in cases of diabetes mellitus. It was also said to act very happily by internal administration in cases where there was a tendency to ammoniacal change in the urine in chronic cystitis. It has been found, however, that when persistently taken dyspeptic symptoms are very apt to intervene, and that care must be observed in its administration. It is eliminated from the body by the kidneys unchanged, and the fæces of patients taking saccharine do not ferment easily, nor does putrefactive changes set in as soon as in other cases. There can be no question regarding its antiseptic qualities; but the *modus operandi* has not as yet been determined. The most plausible explanation is that it forms an unsuitable culture media. It is said to be non-fermentable, and has the property of preventing lactic acid fermentation; also prevents the inversion of starch into glucose, and therefore forms a good addition to our tooth powders and washes.

### PHYSICAL APPEARANCE AND CHARACTERISTICS.

Saccharine is a pearly white powder of a low crystalline structure belonging to the monoclinic system. It is slightly soluble in cold water, more soluble in hot, and freely so in boiling water; but it is precipitated again upon cooling. When in solution it gives an intensely sweet taste, which is not perceived so distinctly when the powder is placed upon the tongue. The solution is strongly acid, and by neutralizing it with potassium hydrate or carbonate the fluid may be made to take up a larger per cent. of the drug, but which separates again upon acidulation. Saccharine is freely soluble in alcohol and fairly so in ether. The powder has an odor not unlike bitter almonds, which is intensified by heating. It is 280 times sweeter than cane sugar, having the property of transmitting a distinctly sweet taste to 70,000 times its own bulk of fluid. The English manufacture it in quantities, putting it up in one ounce packages, and furnishing a small horn spoon that will lift about one-half grain, which is all that is needed to sweeten a cup of tea or coffee. Some persons soon tire of its peculiar intensely sweet taste, which seems to grow more and more. This may be because its qualites are more intensified as it is diluted. The addition of saccharine to tooth powders and washes in the strength of $\frac{1}{2}$ grain to the ounce will undoubtedly have a beneficial effect in preventing lactic acid fermentation, and thus have an antiseptic action on decay. There is no question but that it will come more generally into use as an adulteration for sugar. All should, therefore be familiar with the tests for the pure article. We have collected a few tests which we here append.

TESTS FOR SACCHARINE.

"It fuses at about 392° F.; when fused upon platinum-foil or porcelain it emits a distinctly perceptible odor of oil of bitter almonds or essence of mirbane, and finally burns away without leaving a residue; a white residue would be evidence of mineral adulterations.

Four grains of saccharine should render a clear solution when agitated in a test-tube with 2 drachms of concentrated sulphuric acid; upon gently heating this solution it should remain colorless; an ensuing brown or darker color would indicate an admixture of sugars or other organic adulterants.

An additional test for the addition of grape-sugar consists in dissolving 4 grains of the saccharine in 1 drachm of officinal liquor potassæ, which should remain colorless upon heating for 15 minutes. The same solution, when mixed with $\frac{1}{2}$ drachm of *Fehling's* test solution and heated, should not render a brick-red turbidity; else grape or milk sugar is indicated."

TESTING SUGARS FOR SACCHARINE.

"In order to examine common sugar for an admixture of saccharine, respectively a substitution of cane sugar by grape sugar sweetened with saccharine, the following simple test is recommended: A test-tube, pointed and open at the lower end (a glass syringe answers best), containing about 1 fluid ounce, is closed at the lower orifice by a piece of cork or rubber and is filled with the coarsely powdered sugar to be tested; the tube charged with the sugar is then filled with ether and is left standing for about one hour; then the lower orifice is opened and the ether allowed to flow off into a small porcelain capsule; by placing this on a moderately warm place the ether evaporates, leaving any traces of saccharine behind, which is best and very perceptibly recognized by its extremely sweet taste. It may further be recognized by fusing in the capsule containing the ether residue, at a very gentle heat, a few grains of a mixture of 6 parts of sodium carbonate and 1 part of potassium nitrate; subsequently the fuse is heated to redness. The residue, when cold, is dissolved in a little distilled water, and the filtered solution is tested with a few drops of test-solution of barium chloride. An ensuing white turbidity of barium sulphate would prove the presence of saccharine, which forms at such incineration sulphuric acid, whilst no other sulphates possibly contained in the sugar would be extracted by ether."

